# Case report of canine discoid lupus erythematosus in Guatemala

**DOI:** 10.1002/vms3.1264

**Published:** 2023-12-01

**Authors:** Estefany De León‐Robles, Mariana Colmenares, Gylari Mhelanya Calderón

**Affiliations:** ^1^ Instituto de Investigaciones Químicas, Biológicas Biomédicas y Biofísicas, Universidad Mariano Gálvez. 3a Avenida 9‐00 zona 2, 01002, Interior Finca El Zapote Ciudad de Guatemala Guatemala; ^2^ Clínica veterinaria Ortovet de Guatemala Guatemala City Guatemala

**Keywords:** canine cutaneous lupus, discoid lupus erythematosus, histopathology of canine lupus

## Abstract

An 11‐year‐old male Golden Retriever was presented for consultation due to a chronic progressive lesion on the nose that had started a year before. The majority of the nasal mucosa was affected, with the disruption of the normal architecture, pigment atrophy and abundant peeling on the rostral plane. Histopathology revealed a band of lichenoid infiltrate at the interface and vacuolation of the cells in the basal layer consistent with a diagnosis of canine discoid lupus erythematosus.

## Graphical abstract

Geriatric Golden Retriver was brought to the clinic for a large lesion that involves the nasal plane with atrophic characteristics. The histopathological investigation evidences lymphocytic interphase dermatitis, with basal keratinocyte degeneration, loss of basal cells and basement membrane thickening. This lesion was congruent with discoid lupus erythematosus. The case was approached with topical tacrolimus and azathioprine as immunosuppressant therapeutic, and avoidance to sun exposure was recommended for at least 3 months. Therapeutic protocol was fulfilled for no more than 2 weeks, and no improvement was achieved.

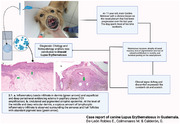

## 1 INTRODUCTION

Canine discoid lupus erythematosus (CDLE) is a chronic, autoimmune disease. The illness in dogs was first described in 1965, but it was not until the 1970s that cutaneous variants were reported in dogs that had developed chronic dermatitis after prolonged exposure to the sun (Griffin et al., 1979; Lewis et al., 1965). The lesions can be confined to the face (facial CDLE) or can be widespread (generalized CDLE) Olivry et al (1987) established the histopathologic classification of CDLE. Histopathology of CDLC revealed similarities with the microscopic lesions present in human cutaneous lupus erythematosus (Griffin et al., 1979). In some Latin American countries, it is still commonly underreported and underdiagnosed due to the low incidence and the lack of widespread knowledge regarding the disease (Farfán‐Arbizú & Chávez‐López, 2020; Henrique et al., 2012; Jiménez, 2008; López & Nadia, 2018). Herein we report the first known case of facial CDLE in Guatemala.

## 2 ANAMNESIS AND CLINICAL EXAMINATION

An 11‐year‐old, male, entire Golden Retriever, weighing 21.4 kg, was evaluated for a chronic lesion on the nasal planum that had been progressively growing over the last year. Weekly shampooing with chlorhexidine over the last 2 months failed to result in any improvement. The owners indicated that the dog spent most of his time outdoors.

## 3 CLINICAL FINDINGS

**FIGURE 1:**
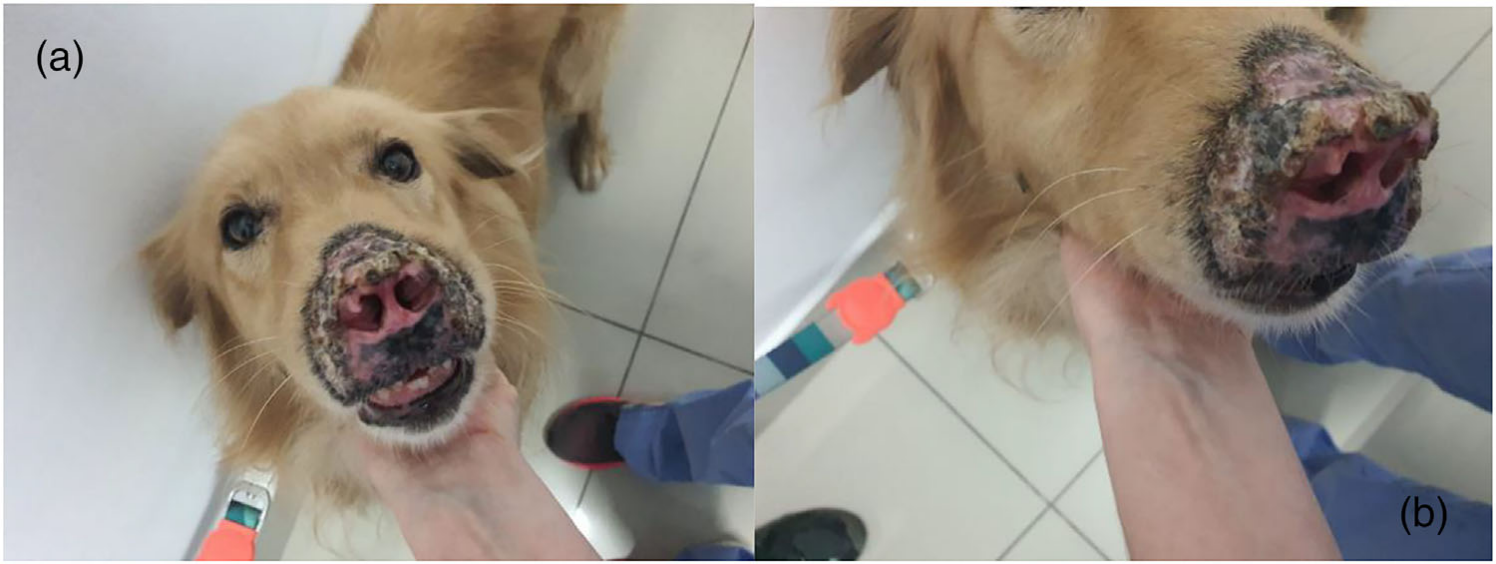
Evidence of lesion in the nasal plane of the patient: (a) strong atrophy of the nasal structure and pigment loss of the area; (b) extension of the lesion occupying the total of the nasal plane and part of the rostral area, scabs and peeling.

Physical examination revealed the atrophy of the nasal mucosa, the distortion of the nasal architecture (Figure 1a), the loss of mucosal pigmentation and the abundant peeling of the nasal planum (Figure 1b). The incisor teeth were absent with associated gum atrophy. The lesion was causing local discomfort resulting in constant rubbing and scratching of the face. The dermal lesions were limited to the nose, and the remainder of the routine clinical examination was unremarkable.

## 4 DIFFERENTIAL DIAGNOSIS

Based on the clinical characteristics, infectious diseases (cutaneous leishmaniasis, fungal, bacterial or mycobacterial infection), allergic dermatitis, neoplasia (cutaneous lymphoma, mast cell tumour and squamous cell carcinoma) and autoimmune diseases (erythema multiforme, mucocutaneous *pemphigus*, epidermolysis bullosa and CDLE) were considered.

## 5 INVESTIGATIONS

A complete blood count and serum biochemistry revealed no clinically significant abnormalities.

Thoracic radiography identified no pulmonary lesions. Cytology of deep skin scrapes from the nasal planum revealed a large number of acantholytic cells and lymphocytic infiltration with no other notable findings.

**FIGURE 2:**
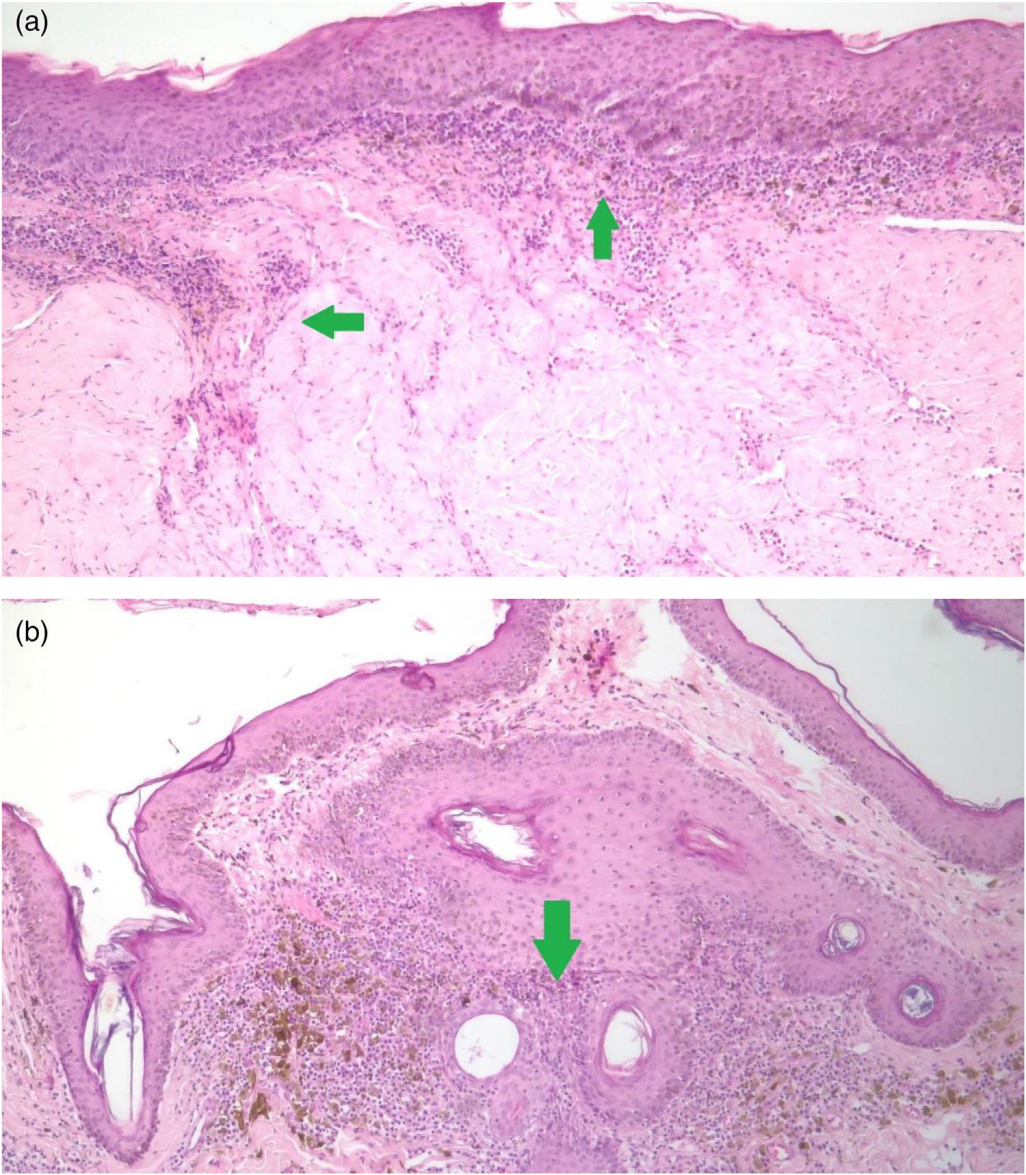
Histological sections analysed with H&E and periodic acid‐Schiff (PAS) techniques. (a) Inflammatory bands infiltrate in dermis (green arrows) and superficial and deep periadnexal evidencing oedema in papillary plexus (10× amplification). (b) Undulated and pigmented atrophic epidermis. At the level of the middle and deep reticular dermis, a copious amount of lymphocytic inflammatory infiltrate is observed surrounding the annexes and hair follicles with abundant pigment loss (green arrow).

**FIGURE 3:**
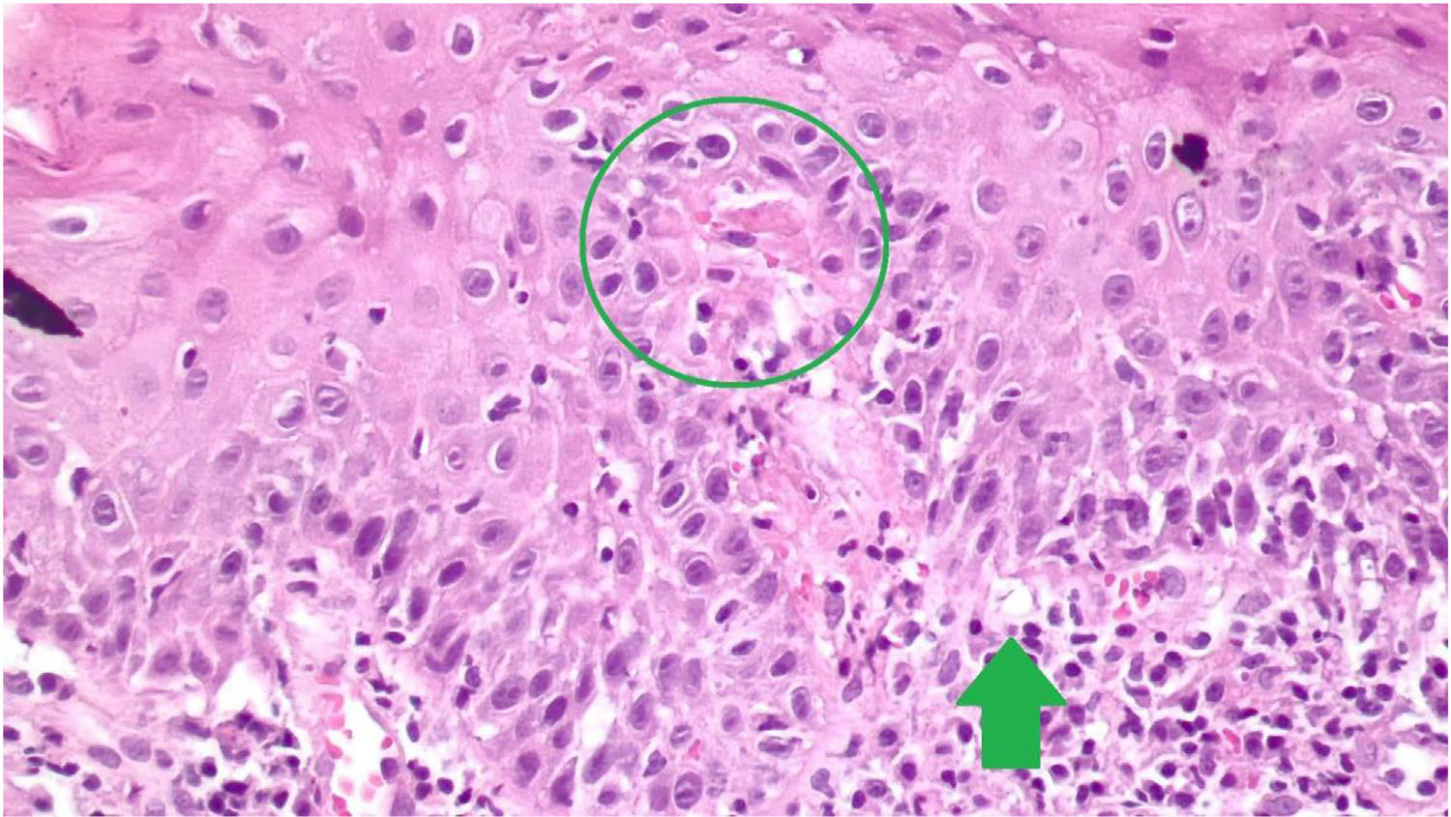
At the green circle, marked vacuolation of the basal layer with diffusing at the dermo‐epidermal junction due to the lichenoid infiltrate (40× amplification). At the green arrow, a prominent Civatte bodies.

**FIGURE 4:**
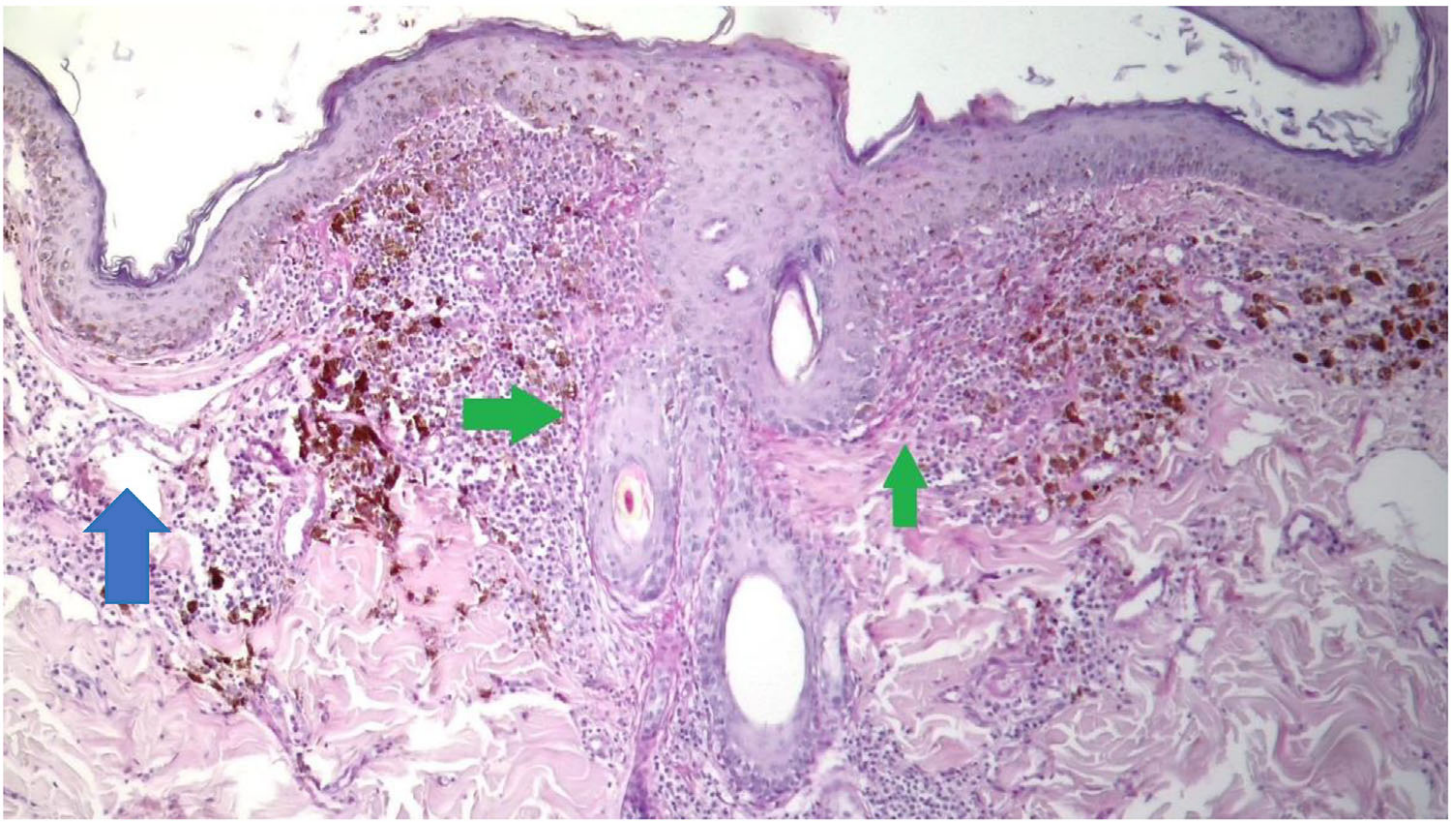
At green arrows, thickening of the basal layer of epidermis and hair follicle and lymphocytic inflammatory cells evidenced by periodic acid‐Schiff (PAS). At blue arrow, detachment at the dermo‐epidermal junction due to the prominent vacuolization of the epidermis basal layer.

**FIGURE 5:**
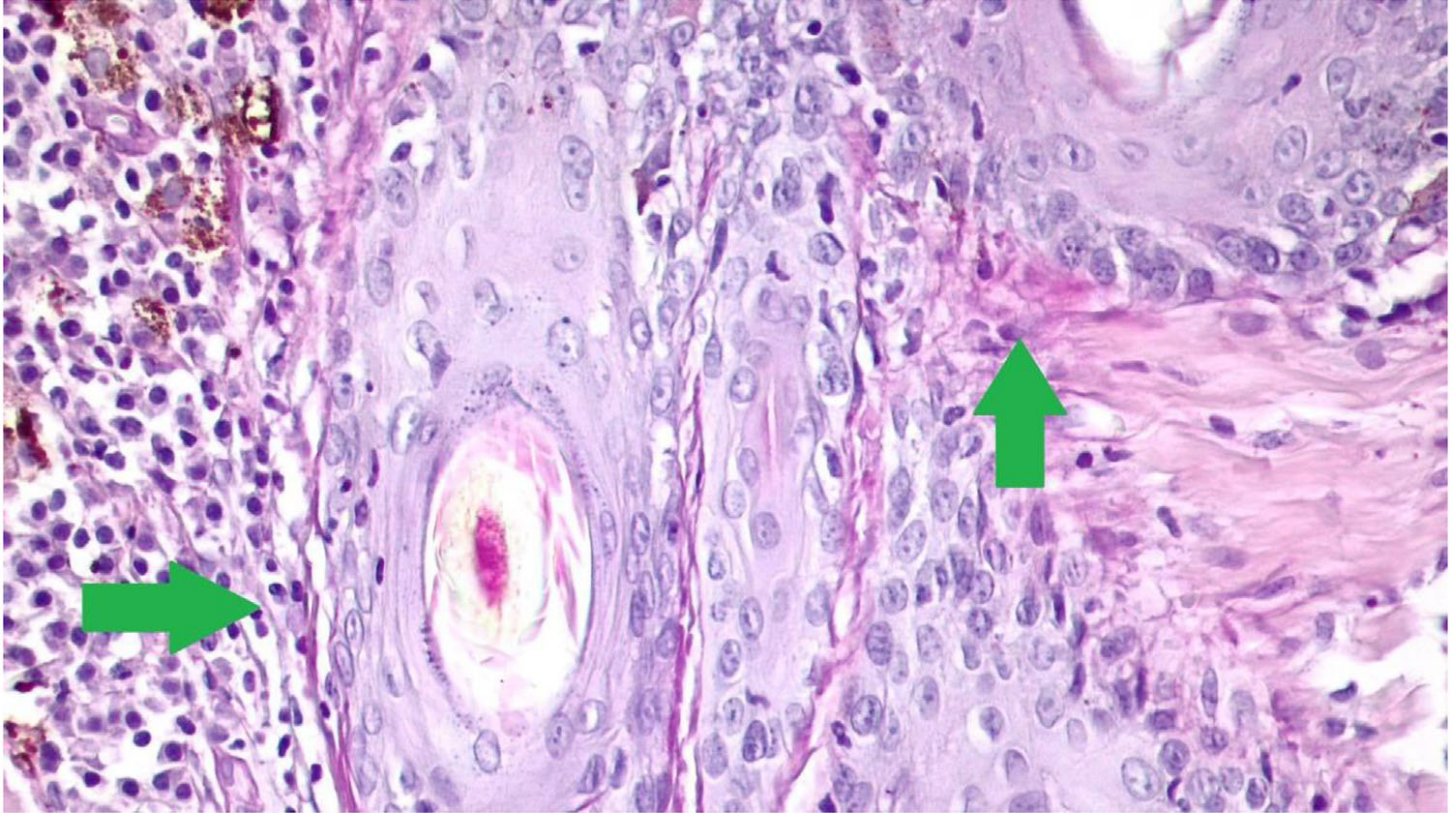
At green arrows, thickening of the basal layer of the hair follicle and thickening of epidermis basal layer highlighted observed with higher magnification.

**FIGURE 6:**
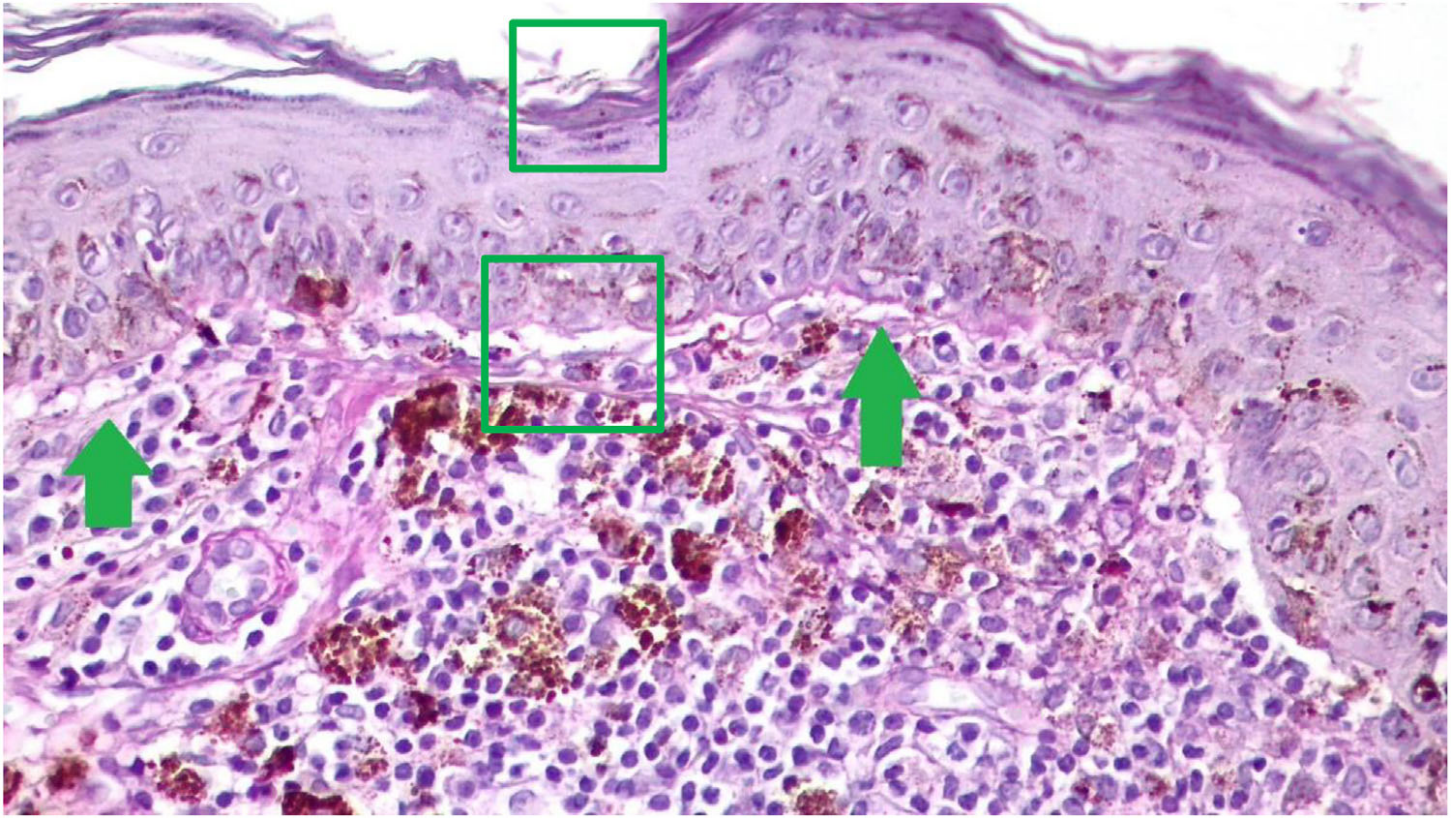
At green square, hyperkeratosis and parakeratosis of the stratum corneum and vacuolated basal layer with a marked separation of the interphase. Inflammatory lymphocytic lichenoid infiltrates at green arrows.

A biopsy was taken with a 4 mm perforator, and the sample was sent in 10% formalin for histopathology analysis. Serial histological sections (Figure 2) revealed atrophic focal areas with mild spongiosis and oedema in the papillary plexus (Figure 2a), associated with marked pigment loss (Figure 2b). Occasional Civatte bodies were present (Figure 3). Vacuolation and thickening of the basal layer were observed and confirmed with the periodic acid‐Schiff (PAS) technique (Figure 3). From the papillary to the deep reticular dermis, an abundant perivascular, periadnexal and perifollicular inflammatory infiltrate was observed (Figures 4, 5 and 4, 5). Hyperkeratosis and parakeratosis were present in circumscribed areas of the epidermis (Figure 6). This infiltrate comprised lymphocytes, histiocytes and plasma cells. Extracellular diploid cocci bacteria were observed in low amounts.

## 6 DIAGNOSIS

The microscopic and clinical characteristics were consistent with facial CDLE (Graphical abstract).

## 7 TREATMENT

Treatment comprised azathioprine (one 50 mg tablet PO every 24 h), vitamin E (one 400 IU/268 mg tablet PO every 24 h) and tacrolimus (applied topically once a day to affected areas) for at least 3 months. Finally, avoiding sun exposure was recommended.

## 8 OUTCOME

The dog was reassessed 3 weeks following the diagnosis. The owners reported that the medical treatment had been administered consistently during the first 2 weeks, but azathioprine had then been discontinued due to vomiting. Avoidance of sunlight had not been possible. Re‐evaluation revealed further deterioration, and the dog appeared to be more uncomfortable. The owners decided not to continue treatment and opted for euthanasia.

## 9 DISCUSSION

CDLE is an autoimmune condition with an unclear aetiology. Available reports are insufficient to conclude the epidemiology of the disease in dogs (Olivry et al., 2018), and further studies are required to provide a better understanding of the pathogenesis, prognosis and optimal treatment (Banovic, 2019; Bryden et al., 2005). Predispositions in the German Shepherd Dog suggest a genetic component (Olivry et al., 2018).

DLE is classified as facial DLE (FDLE) or generalized DLE (GDLE) (Banovic, 2019). The most common presentation in dogs is a single chronic skin lesion, and the presentation in this patient was typical for FDLE which preferentially affects the nasal planum (Olivry et al., 2018). The typical lesions associated with FDLE are erythema, depigmentation, ulceration, crusting and a partial or complete loss of the normal nasal planum with atrophy and cobblestone appearance. In GDLE, the lesions can be generalized or multifocal and are typically distributed over the neck, dorsum and lateral and ventral thorax (Banovic, 2019).

In FDLE, diagnostic imaging typically reveals locally destructive nasal changes with local soft tissue swelling and lytic bone lesions (Banovic et al., 2016). In this patient, thoracic imaging was performed to rule out pulmonary fungal infection or metastasis in the event of infectious or neoplastic disease, respectively. Additional imaging of the head would have helped to define the extent of local disease but was not possible due to lack of owner consent.

Histopathology is the gold standard for differentiation of disease subtypes demonstrating perivascular, superficial, periadnexal and deep microscopic lesions (Kuhn & Landmann, 2014). Correlation between clinical signs and characteristic microscopic findings is required for a definitive diagnosis (Walling & Sontheimer, 2009).

According to Ackerman's algorithm, chronic DLE lesions in humans are typified by interface dermatitis with vacuolation and thickening of the basal layer, a predominant lymphocytic infiltrate, epidermal thinning and diffuse granulomatous infiltration (Ackerman, 1997) (Table 1). Consistent with this, the histopathology of the skin biopsy from our patient showed a lichenoid infiltrate in a band at the dermo‐epidermal junction and vacuolation of cells in the basal layer. These lesions are congruent with those of generalized CDLE reported by Banovic et al. (2016).

**TABLE 1:** Histopathological analysis of the biopsy.

Lesion location	Description of the injury	Most observed	Observed in this patient
Epidermis	Basal plane vacuolization	+	+
	Orthokeratosis and parakeratosis in the stratum corneum	++	+
	Basement membrane hyperplasia and hyperkeratosis of the stratum corneum	+++	++
Dermal–epidermal junction (interface)	Interface with loss of colour and lichenoid infiltration in the basement membrane of the epidermis	+++	+++
Dermis	Perivascular dermatitis with lymphoid infiltration	+++	+
	Oedema of the papillary plexus and abundant mucin in the cutaneous plexus (reticulated dermis)	+	−
	Satellite lymphocytes to apoptotic keratinocytes, deposited in the basement membrane of the epidermis	+	−
Follicular and perifollicular	Perifollicular inflammatory infiltrate	++	++
	Loss of pigment in the follicular epithelium	+	−

*Note*: Principal canine discoid lupus erythematosus – CDLE – microscopic lesions and frequency. Observance level: +++, most observed; ++, medium observed; +, less observed; −, no observed.

*Source*: Ackerman (1997) and Banovic (2019).

The diagnosis of FDLE was considered to be sufficient based on the anamnesis, clinical examination and histopathologic results; thus, confirmatory tests were not requested. Although serum antinuclear antibody testing is extremely useful in the diagnosis of systemic lupus erythematosus (SLE), positive results are rare in patients with DLE; positive results are found in only ∼5% of DLE patients compared with 85%–90% of patients with SLE (Banovic, 2019; Bedolla et al., 2009). The diagnosis of DLE can be further supported by direct immunofluorescence and immunohistochemistry techniques, with 50% of patients demonstrating continuous and linear IgG deposition within plasma cells of the superficial dermis (Balazs‐Mayanz & Nolasco‐Espinosa, 2017). However, in Guatemala, these techniques are available only in research laboratories.

Current literature states that the specific lesions of CDLE are grouped on the basis of histology, duration of lesions, laboratory abnormalities and clinical findings (Olivry et al., 2018). The clinical findings and histopathologic analysis of the dog described here were consistent with FDLE, and with a reported exposition in large periods to the sun and duration of lesions >12 months, this would be considered a chronic case (Banovic, 2019).

The CDLE pathogenesis of CDLE remains under study but is considered multifactorial; genetics, environmental triggers and innate adaptive immune response all play a part (Hejazi & Werth, 2016). Consideration of this complex pathogenesis is required to achieve effective treatment strategies, which should ideally include environmental modification as well as drug therapy (Hejazi & Werth, 2016). In particular, it is known that ultraviolet light induces a cytokine inflammatory cascade that eventually causes apoptosis of skin cells, thus contributing to DLE lesions (Hejazi & Werth, 2016). It is important that patients with DLE are protected against harmful ultraviolet rays but, as illustrated in this patient, the avoidance of sun exposure is not always possible. Although sunscreen can be used, it has the disadvantage of being easily licked off by dogs (Balazs‐Mayanz & Nolasco‐Espinosa, 2017).

Currently, as in humans, FDLE in dogs does not have a specific treatment approved (Harvey et al., 2023). It is known that CDLE is a consequence of hyperactive innate and adaptive immune systems; thus, various immunosuppressive and immunomodulatory treatments have been tried various immunosuppressive and immunomodulatory treatments have been tried both topically and systemically (Olivry et al., 2018).

Immunosuppressive doses of oral prednisolone are commonly prescribed as first‐line treatment due to the drugs’ accessibility and cost. However, responses are variable and prolonged use is linked to side effects (Olivry et al., 2018). Other immunosuppressive drugs, such as cyclosporine (4.8 mg/kg PO twice daily) or azathioprine (1.5 mg/kg PO once daily), have been recommended due to their safety and effectiveness (Font et al., 2006; Jackson, 2004). Topical steroidal cream is also encouraged for local immunosuppression, usually with betamethasone 0.1%. However, it can also be problematic if rubbed or licked off (Banovic, 2019; Walling & Sontheimer, 2009). Additionally, immunomodulatory drugs such as tetracycline and niacinamide have been utilized (Adolph et al., 2014); however, response rates to these treatments have been mixed. White et al. (1992) found that tetracycline and niacinamide resulted in a good‐to‐excellent response in 70% of treated dogs, and a recent retrospective study reported a similar response rate of 67%, but with a high recurrence rate (Banovic et al., 2016). The mechanism behind the synergy and mode of action of these drugs remains unclear (Adolph et al., 2014). For chronic cases, oral vitamin E (no more than 400 IU per day until remission) has been advocated as a skin regenerator (Olivry et al., 2018).

According to Hejazi and Werth (2016), treatment for humans with lesions should follow three stages: prevention (minimizing sun exposure), first‐line treatment (using topical steroids or calcineurin inhibitors) and second‐line treatment (using systemic steroids or other immunosuppressants). Although we attempted to follow this approach in this case, the owners were unable to significantly reduce sunlight exposure, and systemic immunosuppression with azathioprine was not tolerated. This ultimately led to a suboptimal treatment strategy which is likely to have contributed to the poor outcome. With regards to this, it is important to note that side effects are commonly seen with immunosuppressant medications, such as calcineurin inhibitors, steroids and thiopurines, and that these drugs often require at least 10 weeks to improve the remission of lesions (Hejazi & Werth, 2016; Olivry et al., 2018). The first aim of FDLE treatment should therefore be to educate owners on the chronic nature of this disease, the importance of avoiding potential triggers or exacerbating influences and the importance of long‐term medication compliance (Hejazi & Werth, 2016).

CDLE may result in significant disfigurement and discomfort, leading to the poor quality of life (Hejazi & Werth, 2016). Since the time of the first examination, extension and progression of the lesion were reported to be rapid, and feedback from the owners indicated that this had become a significant source of discomfort. The patient had not received analgesia, which would have compounded his poor quality of life and may also have influenced the owner's willingness to continue treatment. This highlights the importance of discussing and formulating comprehensive analgesia plans in patients with chronic progressive disease.

New and more effective treatment options for CDLE are becoming available. In chronic DLE, inflammatory proteins have been identified as a primary cause of the disease. Recent studies suggest that the skin lesion transcriptomes of both canine and human CLEs are similar, with both showing activation of the innate immune system, interferon and JAK (Janus Kinase)‐STAT (Amudzi et al., 2022; Harvey et al., 2023). An experimental study has shown excellent responses in the treatment of FDLE cases, with improvement observed within the first 2 weeks of using oclacitinib at 1.8 mg/kg twice daily. Total remission was achieved at 4 weeks of treatment (Harvey et al., 2023). Oclacitinib is a nonselective JAK inhibitor that targets the primary cause of this disease. Although this protocol is still under research, the recent findings could help to improve patient outcomes more quickly.

## 10 CONCLUSION

Canine dermatological conditions are common in Guatemala, and CDLE is a condition reported in many countries. This is the first report of a clinical case of CDLE observed in Guatemala City. It is likely that the lack of cytologic and/or histopathologic information in many patients has resulted in the underdiagnosis of this condition.

This case report aims to increase the awareness of CDLE in dogs in Guatemala, to ensure that veterinarians consider it as part of their differential diagnosis, and take this into consideration in their clinical decision‐making.

It is also crucial to highlight the importance of interdisciplinary communication between the veterinarian and pathologist in order to confirm that the microscopic findings are consistent with the clinical findings. Documentation of pertinent information from all clinical cases is essential for sharing clinical perspectives and enabling further education of veterinarians regarding the approach and benefits of diagnostic testing as opposed to blind treatment trials.

## AUTHOR CONTRIBUTIONS

*Conceptualization; formal analysis; investigation; writing—original draft*: Estefany De León‐Robles. *Data curation*: Mariana Colmenares. *Data curation; formal analysis; supervision; validation*: Gylari Mhelanya Calderón.

## CONFLICT OF INTEREST STATEMENT

All authors agree to declare that they have no conflicts of interest.

### PEER REVIEW

The peer review history for this article is available at https://www.webofscience.com/api/gateway/wos/peer‐review/10.1002/vms3.1264.

## ETHICS STATEMENT

Ethical approval was not required for this paper.

## FUNDING INFORMATION

No fundings was used to this paper.

## Data Availability

Data are available on request from the authors.
